# Transcriptomic and Phenotypic Analyses Reveal the Molecular Mechanism of Dwarfing in Tetraploid *Robinia pseudoacacia* L.

**DOI:** 10.3390/ijms25021312

**Published:** 2024-01-21

**Authors:** Yue Wu, Qi Guo, Cui Long, Yousry A. El-Kassaby, Yuhan Sun, Yun Li

**Affiliations:** 1 State Key Laboratory of Tree Genetics and Breeding, Engineering Technology Research Center of Black Locust of National Forestry and Grassland Administration, National Engineering Research Center of Tree Breeding and Ecological Restoration, College of Biological Sciences and Technology, Beijing Forestry University, Beijing 100083, China; wuyue55@bjfu.edu.cn (Y.W.);; 2 Department of Forest and Conservation Sciences Faculty of Forestry, The University of British Columbia, 2424 Main Mall, Vancouver, BC V6T 1Z4, Canada; y.el-kassaby@ubc.ca

**Keywords:** *Robinia pseudoacacia* L., autotetraploid, dwarfism type, transcriptome, plant circadian rhythm

## Abstract

Polyploid breeding techniques aid in the cultivation of new forestry cultivars, thus expanding the suite of strategies for the improvement of arboreal traits and innovation within the field of forestry. Compared to diploid *Robinia pseudoacacia* L. (black locust) ‘D26-5①’ (2×), its dwarfed homologous tetraploid ‘D26-5②’ (4×) variety has better application prospects in garden vegetation guardrails and urban landscape. However, the molecular mechanism of the generation and growth of this dwarf variety is still unclear. Here, plant growth and development as well as histological differences between the diploid and its autotetraploid were investigated. Levels of endogenous hormones at three different developmental stages (20, 40, and 70 days) of 2× and homologous 4× tissue culture plantlets were assessed, and it was found that the brassinosteroid (BR) contents of the former were significantly higher than the latter. Transcriptome sequencing data analysis of 2× and homologous 4× showed that differentially expressed genes (DEGs) were significantly enriched in plant hormone synthesis and signal transduction, sugar and starch metabolism, and the plant circadian rhythm pathway, which are closely related to plant growth and development. Therefore, these biological pathways may be important regulatory pathways leading to dwarfism and slow growth in tetraploids. Additionally, utilizing weighted gene coexpression network analysis (WGCNA), we identified three crucial differentially expressed genes (DEGs)—*PRR5, CYP450*, and *SPA1*—that potentially underlie the observed ploidy variation. This study provides a new reference for the molecular mechanism of dwarfism in dwarfed autotetraploid black locusts. Collectively, our results of metabolite analysis and comparative transcriptomics confirm that plant hormone signaling and the circadian rhythm pathway result in dwarfism in black locusts.

## 1. Introduction

Polyploid breeding is a crucial approach for genetic improvement in plants as it leads to significant alterations in the genetic traits and phenotypes of plants through chromosome doubling [1]. This process provides a broader scope for genetic variation, facilitating the development of new varieties with superior characteristics and thereby enriching the repertoire of strategies available for the amelioration of tree characteristics and forestry innovation [2,3]. Polyploid plants obtained through conventional polyploid breeding exhibit an increase in the number of gene copies and alterations in gene expression regulation. Consequently, this can potentially impact various aspects of plant growth, including growth rate, height, and morphology [4]. Moreover, based on phenotypic characteristics, polyploid plants derived from conventional polyploid breeding can typically be classified into two categories, namely, rapid growth giant and dwarf types [5]. Among them, the rapid growth giant type is more common, which usually shows fast and robust growth, huge organs, and high resistance to drought and salt [6]. Another category of stunted polyploids, characterized by their typically sluggish growth and shorter stature compared to their conventional diploid counterparts, often receive scant attention or are prematurely discarded in initial rounds of selective breeding programs [7]. However, with the diversification of seedling variety demands in recent years, these dwarf traits will improve the agronomic or ornamental value of varieties. Therefore, dwarf polyploids have been gradually exploited and utilized, becoming a new breeding direction [8].

Plant polyploidy causes changes in gene expression patterns, which further affects plant growth and development [9]. Through pathway analysis of tetraploid and diploid differentially expressed genes (DEGs) in *Arabidopsis thaliana*, it was found that these genes were mainly enriched in photosynthesis and metabolism pathways [10]. *A. thaliana* allotetraploids grow faster and more robust than their diploid counterparts. Differential gene expression analysis showed that plant circadian clock regulators, such as *LHY* and *CCA1*, were downregulated, thus upregulating the expression of downstream genes involved in chlorophyll synthesis and starch metabolism [11,12]. Compared to diploids, *populus* triploids have the advantages of fast growth and strong resistance, and through DEG analysis, it was found that the upregulated genes were mainly concentrated in processes related to compound metabolism, antioxidants, and cell growth [13]. Plant endogenous hormones are trace organic compounds that autonomously regulate their own growth and development, encompassing auxin, gibberellin (GA), cytokinin (CTK), abscisic acid (ABA), ethylene (ETH), and brassinosterol (BR) [14]. The change of endogenous hormone content will affect plant growth and development [15]. At the same leaf position, genes related to photosynthesis- and chlorophyll-related processes were upregulated in triploid *populus*. Similarly, genes related to the synthesis and transduction of hormones, such as auxin, mitogen, and brassinolide, were also upregulated, which led to speculation that the molecular mechanism that enhances photosynthesis and possible synthesis and transduction of plant hormones was the reason for its fast growth [16,17,18]. Liao et al. [19] also found that after chromosome doubling, hormones such as auxin, CK, and GA increased in *populus*. Studies on diploid and tetraploid mulberry (*Morus alba*) showed that the tetraploid had growth advantages over the diploid, and their DEGs were significantly enriched in plant hormone synthesis and signal transduction pathways, indicating that they may be the reason for the faster growth of tetraploids [20]. After chromosome doubling, the endogenous hormone content of *Hybrid sweetgum* changed correspondingly, thus leading to growth and development variation of polyploid plants [21]. In willows (*Salix*), it was found that endogenous mitogen, GA, and SA contents also increased after chromosome doubling [22].

However, after polyploidy, some plants suffer from phenotypic disadvantage and slow growth and become dwarfed, such as triploid papaya [23] and polyploid ginger [24]. Tetraploid wheat had higher leaf area and chlorophyll content than diploid but a lower photosynthetic rate [25]. Tetraploid potato’s leaf area was significantly larger than that of its diploid counterpart [26]. Studies have shown that tetraploid apples were significantly stunted and dwarfed compared to their diploid counterparts with inferior phenotypic traits, such as slow growth and short plants [5]. Differences between polyploid and diploid *Citrus limonia* were mainly concentrated in water stress and growth regulation processes, which were speculated to be the pathways related to growth and development [27]. By comparing tetraploid and diploid apple, it was found that the downregulated genes in the tetraploid were mainly involved in the metabolism of auxin and BR pathways, so it was speculated that the plant hormone signal transduction pathway may be an important regulatory pathway affecting the slow and short growth of apple tetraploid [9].

While *Robinia pseudoacacia* L. (black locust) is considered an invasive species in certain European countries, it is undeniable that it possesses a significant ecological and economic value, such as in ornamental horticulture, timber, and feeding [28]. In particular, black locust has a good application prospect in the fields of garden vegetation guardrail and urban landscape afforestation [29,30]. In these fields, it has a good potential for exploitation due to its strong stress resistance and easy maintenance [31]. Both diploid and tetraploid varieties have been identified in black locusts, exhibiting distinct physiological characteristics [32,33]. However, the causes of dwarfism in tetraploid black locust plants remain unknown. It should be noted that the growth and developmental differences between diploid and homoeologous tetraploid are not absolute as these differences may be influenced by other factors, such as environmental conditions, gene interactions, and genetic backgrounds [34]. In this study, the phenotypic and cytological differences between diploid and autotetraploid black locust plants were compared, and transcriptome sequencing was performed to analyze the causes of dwarfism of the tetraploid plants at the molecular level. In addition, the contents of endogenous hormones at different developmental stages of the two plant varieties were measured so as to further analyze the molecular mechanism of dwarfism in tetraploid black locust plants.

## 2. Results

### 2.1. Ploidy Identification

**Figure 1:**
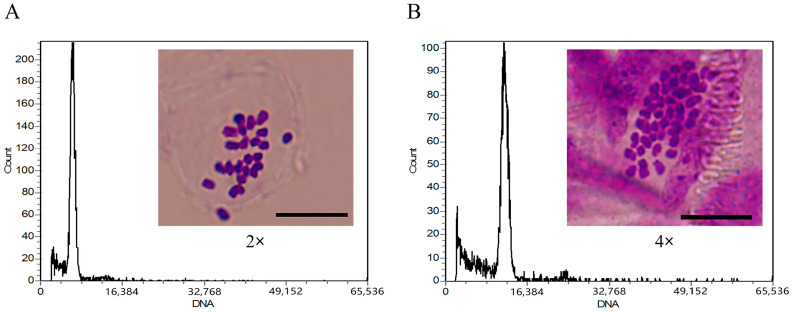
Histograms of flow cytometry and chromosome count for the diploid ‘D26-5①’ (2n = 2× = 22) (**A**) and autotetraploid ‘D26-5②’ (2n = 4× = 44) (**B**) of black locust (bar = 10 µm).

The content of DNA in fresh young leaves of diploid ‘D26-5①’ and autotetraploid ‘D26-5②’ black locust plants was determined by flow cytometry with diploid leaves as control. The results showed that the position of the relative fluorescence main peak of the ‘D26-5①’ plant was consistent with that of the control, and the position of the relative fluorescence main peak of the ‘D26-5②’ plant was twice that of ‘D26-5①’, confirming its true ploidy status (Figure 1).

To further determine the ploidy of ‘D26-5①’ and ‘D26-5②’, the shoot tips of their potted plantlet were collected for a chromosome count, and it was found through microscopic observation that their chromosome number corresponded to their ploidy stats (i.e., ‘D26-5①’ plants had 2n = 2× = 22 and ‘D26-5②’ plant had 2n = 4× = 44) (Figure 1A,B).

### 2.2. Morphological and Physiological Characteristics of Plants

**Figure 2:**
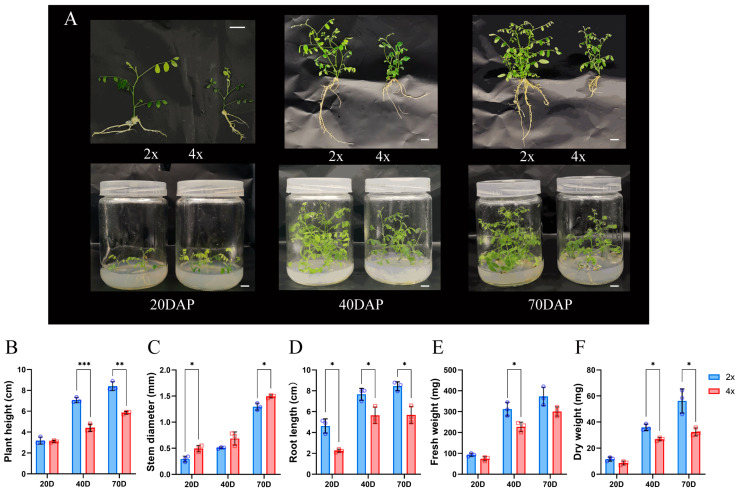
Comparison of phenotypic indexes for 2× and 4× black locust tissue culture plantlets at different developmental stages. (**A**) Plant morphology at 20, 40, and 70 DAP. (**B**) Plant height. (**C**) Stem diameter. (**D**) Root length. (**E**) Fresh weight. (**F**) Dry weight. Scale in (**A**): 1 cm. * represents significant difference (* *p* < 0.05); ** and *** represent highly significant differences (** *p* < 0.01 and *** *p* < 0.001).

Phenotypic comparison between 2× and 4× plants at 20, 40, and 70 DAP indicated that the latter was shorter in height and had a reduced primary root length compared to the former (Figure 2A). Morphologically significant differences began to appear at 40 DAP after subregeneration with 2× plant height 1.60 times that of 4× (Figure 2B). Subsequently, plant height also had a significant difference after six months of transplanting (Figure S1).

The plant height of 2× and 4× during the early stage (20 DAP) was short, and the difference was not significant, but the growth rate of 2× in mid and late stages (40 and 70 DAP) was significantly higher than that of 4× (Figure 2B). Throughout the whole developmental cycle, the stem diameter of 4× was larger than that of 2×, and the stem diameter of 4× was significantly higher than that of 2× at the early and late stages (20 and 70 DAP) (Figure 2C). The 4× root length was consistently shorter than that of 2× throughout the different stages of growth and development (Figure 2D). Altogether, the growth rate of 2× plant height was significantly higher than that of 4×, and the stem thickening rate of 4× was significantly higher than that of 2×. Furthermore, the fresh and dry weights of 4× plants were always lower than those of 2× plants (Figure 2E,F). There was a significant difference in both fresh and dry weights at 40 DAP (fresh and dry weights of 2× were 1.37 and 1.34 times higher than those of 4×, respectively).

### 2.3. Properties Related to Photosynthesis

**Figure 3:**
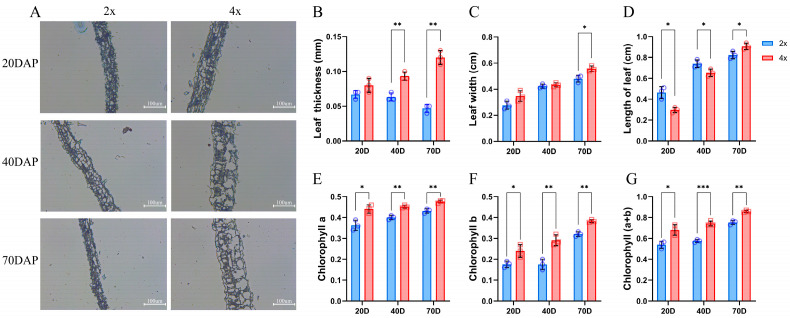
Comparison of morphology and chlorophyll content between 2× and 4× black locust plants at different developmental stages. (**A**) Longitudinal anatomical structure of leaf. (**B**) Length of leaf. (**C**) Leaf width. (**D**) Leaf thickness. (**E**) Chlorophyll-a content. (**F**) Chlorophyll-b content. (**G**) Chlorophyll (a + b) content. * represents significant difference (* *p* < 0.05); ** and *** represent highly significant difference (** *p* < 0.01 and, *** *p* < 0.001).

Because leaf size, chlorophyll content, and stomatal size are important indicators of plant development, we chose the 4th compound leaf of black locust to measure these metrics. The thickness of 4× leaflets was greater than that of 2× leaflets, with a significant difference observed at 40 and 70 DAP, while the difference was not significant at 20 DAP (Figure 3A). The difference in leaflet thickness became more pronounced as the growth time increased, with 4× leaflets being 1.48 times thicker at 40 DAP and 2.57 times thicker at 70 DAP compared to 2× leaflets (Figure 3B). In addition, the 4× leaf width was significantly larger than that of 2× in the late stage (Figure 3C). The leaf length of 4× was significantly larger than that of 2× in the early and middle stages (20 and 40 DAP), and the opposite phenotype occurred at a later stage (70 DAP) (Figure 3D). Based on the results mentioned, it appears that the leaves of 4× developed faster than those of 2× between 40 and 70 DAP. Furthermore, the chlorophyll content (a + b) in 4× leaves was significantly higher compared to that in 2× leaves. This difference grew more significant as the culture time increased, as shown in Figure 3E–G.

**Figure 4:**
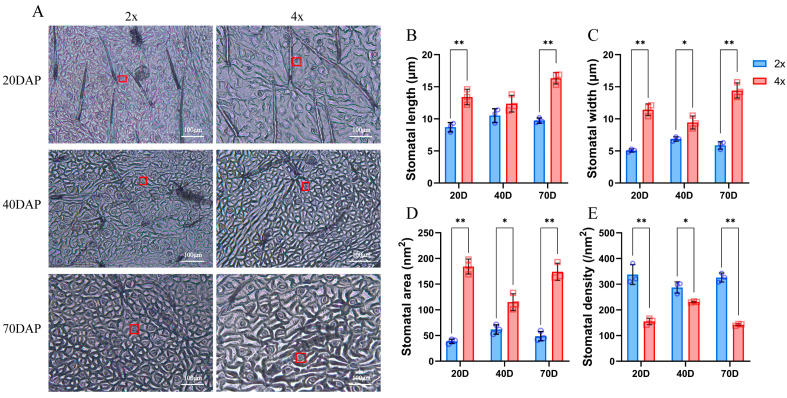
Observation of stomata at different developmental stages of 2× and 4× black locust plants. Histological observations at 20, 40, and 70 DAP (**A**). (**B**) Stomatal length. (**C**) Stomatal width. (**D**) Stomatal area. (**E**) Stomatal density. * represents significant difference (* *p* < 0.05); ** represents highly significant difference (** *p* < 0.01). Red boxes indicate individual stomata.

Stomata size of 4× was significantly larger than that of 2× (Figure 4A–D), while the opposite trend was observed for stomata number, which showed lower density per unit area of leaves (Figure 4E). These results indicate that the stomatal size increased with the increase in ploidy, while the stomatal density decreased with the increase in ploidy.

### 2.4. Endogenous Hormone Content Determination

**Figure 5:**
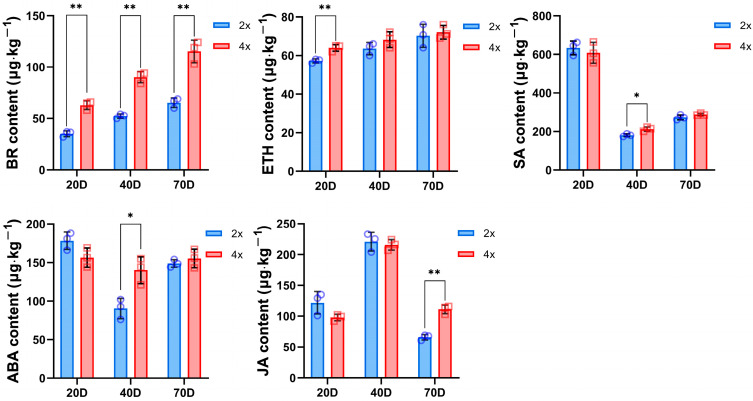
Comparison of endogenous hormone content between 2× and 4× black locusts at different developmental stages. * represents significant difference (* *p* < 0.05); ** represents highly significant difference (** *p* < 0.01).

Based on the previous descriptions, it was observed that 4× had more dwarfed plants compared to 2×. Previous studies have highlighted the significance of plant hormones in influencing growth and development. Therefore, it is plausible to suggest that the observed phenomenon could be attributed to the plant hormone signal transduction pathway [21]. To verify the effect of plant hormones on growth rate, the contents of auxin, gibberellin (GA), cytokinin (CTK), abscisic acid (ABA), ethylene (ETH), brassinosterol (BR), jasmonic acid (JA), and salicylic acid (SA) at different periods of 2× and 4× were determined (Figure 5). The results showed that the BR contents in 4× were significantly higher than those in 2× at the three different growth stages. Similar observations were detected for ETH (at 20 DAP), SA and ABA (at 40 DAP), and JA (at 70 DAP), while no significant difference was observed in the levels of auxin and cytokinins (CTK) (Figure S2). However, no detection of gibberellin content was observed. These results indicate that the high BR, ETH, SA, ABA, and JA contents in 4× may be the reason for the slow growth compared to 2×.

### 2.5. RNA Sequencing and qRT-PCR

To resolve differences at the molecular level, we performed transcriptome analysis of 2× and 4× at different stages. Transcriptome sequencing was performed on 18 samples representing six groups from the three periods (20, 40, and 70 DAP) of 2× and 4× black locusts, with three biological replicates per group. The indicators of Q20, Q30, and GC content were qualified, indicating that the quality of sequencing results was controlled (Table S1).

**Figure 6:**
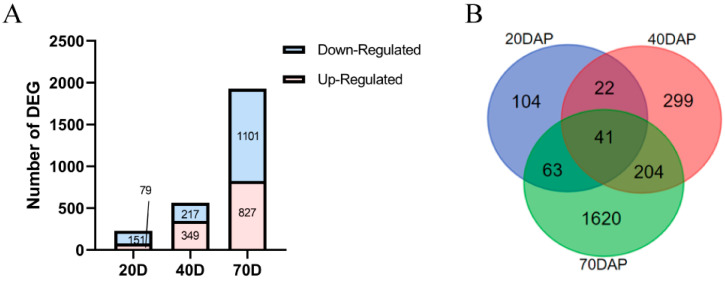
Statistics of differentially expressed genes at different developmental stages (20, 40, and 70 DAP). (**A**) Numbers of differentially expressed genes. (**B**) Venn diagrams.

By comparing the gene expression levels of 2× and 4× at 20, 40, and 70 DAP, a total of 27,571 genes and 2353 differentially expressed genes (DEGs) were detected. There were 230 DEGs between 2× and 4× at 20 DAP, among which 79 and 151 were up- and downregulated, respectively. At 40 DAP, 566 DEGs were detected, among which 349 and 217 were up- and downregulated, respectively. There were 1928 DEGs at 70 DAP, among which 827 and 1101 were up- and downregulated, respectively (Figure 6). The results showed that most of the DEGs were downregulated in 4×, and the number of DEGs increased with the increase in growth time and reached the maximum value at 70 DAP.

A total of 14 DEGs of 2× and 4× were selected for qRT-PCR verification (Table S2). The results demonstrated a high degree of concordance between the gene expression levels obtained from qPCR and FPKM data generated by transcriptome sequencing, thus validating the reliability of the transcriptome analysis for downstream investigations (Figure S3).

### 2.6. Enrichment Analysis of Differentially Expressed Genes

In order to analyze the reasons for the differences in growth rates between 2× and 4× at different growth stages (20, 40, and 70 DAP), GO was used to analyze them from the biological process (BP), cell component (CC), and molecular function (MF) aspects. At 20 DAP, DEGs of 2× and 4× related to plant growth and development mainly included structural components of the cell wall (all downregulated), amino acid transport metabolism, and the hormone metabolic process (Figure S4A). After 40 DAP, DEGs were mainly concentrated in photosynthesis, chlorophyll binding (all upregulated), and starch metabolic process (all upregulated) (Figure S4B). As for 70 DAP, DEGs were mainly concentrated in the cell wall macromolecule metabolic process and the correlation between hormone synthesis and metabolism (Figure S4C).

To further analyze the metabolic pathways involved in DEGs of 2× and 4×, KEGG pathway enrichment analysis was used to analyze their enrichment pathways. After 20 DAP, the enrichment results showed that the differential genes were significantly enriched in zeatin biosynthesis, amino acid metabolism, and carbon fixation in photosynthetic organisms. At 40 DAP, the differentially expressed genes were significantly enriched in chlorophyll metabolism, photosynthesis, the plant circadian rhythm, and secondary metabolic pathways. As for 70 DAP, the differential genes were significantly enriched in plant hormone signal transduction pathways, the plant circadian rhythm, biosynthesis, and transport of secondary metabolites (Figure S5).

Through the analysis of functional enrichment pathways of DEGs of 2× and 4×, the main enrichment pathways were amino acid metabolism and the hormone metabolic process at 20 DAP, photosynthesis and the plant circadian rhythm at 40 DAP, and plant hormone signal transduction and the metabolic process at 70 DAP. These results suggest that the phenotypic differences of 2× and 4× plants may be due to the differences in these metabolic pathways during development.

### 2.7. Weighted Gene Coexpression Network Analysis (WGCNA)

**Figure 7:**
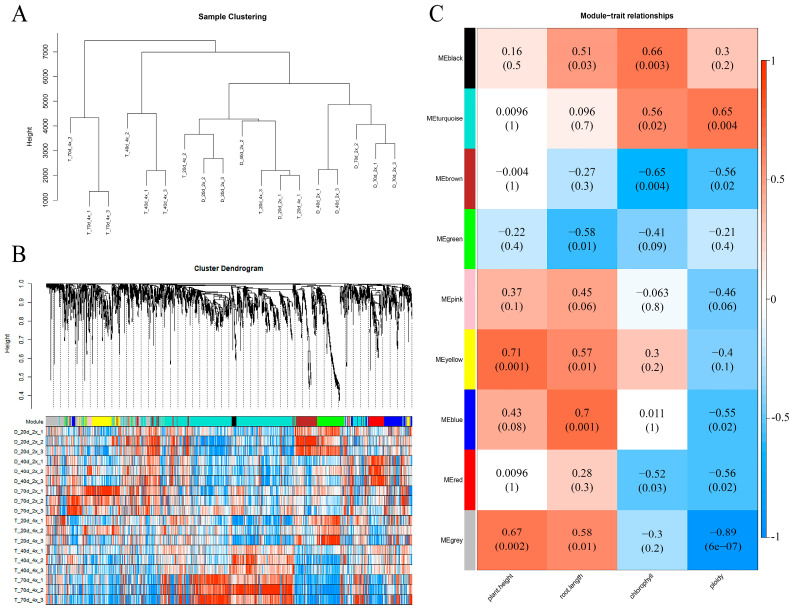
Weighted gene coexpression network analysis (WGCNA). (**A**) Hierarchical cluster analysis of samples. (**B**) Gene cluster tree and module division. (**C**) Correlation between modules and traits.

Hierarchical clustering was performed on the test samples according to the differences in gene expression patterns. Firstly, according to the expression levels (FPKM value) of all 2353 DEGs in 2× and 4× at different development time points (20, 40, and 70 DAP) and after the removal of samples and genes that did not meet the criteria, 18 test samples were clustered after filtering (Figure 7A). The clustering results showed that all samples were basically clustered according to the different growth time points of different ploidy. The 70 DAP of 2× (D-70 d-2×), 40 DAP (T-40 d-4×), and 70 DAP (T-70 d-4×) of 4× were well clustered together separately. However, the 20 DAP of 2× (D-20 d-4×) clustered with the 4× (T-20 d-4×), indicating that the overall expression levels of 2× and 4× genes at 20 DAP were similar with little differences.

According to the gene expression pattern, hierarchical clustering was performed on the DEGs, and the minimum number of modules was set as 30. According to the module characteristic value, the DEG modules of 2× and 4× were further integrated, and a total of nine coexpressed modules with different colors were divided (Figure 7B). Different modules have different gene expression patterns and participate in different biological functions. In order to screen out key modules related to growth and development differences caused by ploidy, different modules associated with plant height, root length, chlorophyll, and ploidy phenotypic traits were analyzed (Table S3). Correlation between gene coexpression modules and the analyzed traits (Figure 7C) indicated that all phenotypic features were significantly correlated with at least one coexpressed module. For example, the yellow, blue, black, and turquoise modules showed significant correlations with plant height (0.71; *p* = 0.001), root length (0.7; *p* = 0.001), chlorophyll (0.66; *p* = 0.003), and ploidy (0.65; *p* = 0.004), respectively. The larger the absolute value of module significance level, the higher the degree of correlation between the module and the phenotype (when *p* was less than 0.05, the relationship was significant). Due to the different ploidy results in different phenotypic characteristics in black locusts, here, we focused on growth and development differences between two kinds of ploidy caused by doubling the chromosome number. More attention was paid to the modules closely related to ploidy in the later period, and the turquoise module was the coexpression module that needed further attention and analysis in the later period.

Through correlation analysis of modules and traits, it was found that the turquoise module was the one most relevant to ploidy with the mode DEGs (999). Through gene GO enrichment analysis of this module, it was found that most of the genes were involved in the metabolic process, cellular process, biological regulation, and catalytic activity of stimulus response. The pathway enrichment analysis of genes showed that most genes were enriched in hormone signal transduction, secondary metabolite synthesis, amino acid metabolism, and other pathways, while genes in the module were significantly enriched in the plant circadian rhythm, brassinosteroid biosynthesis, and the linoleic acid metabolism pathway (Figure S6). These results suggest that the circadian rhythm and plant hormone signal transduction may be the key regulatory pathways for the difference between 2× and 4×.

**Figure 8:**
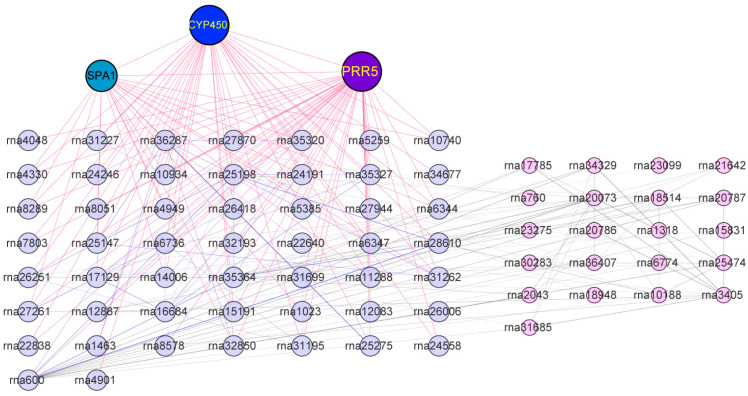
Key regulatory networks in turquoise, a core module closely related to ploidy. Genes depicted in light purple represent direct targets of the hub gene, while those in pink are indirect targets of the hub gene. The red line connects the target genes of the hub gene.

Hub gene is a gene with high connectivity in the module and plays a key regulatory role in the module. It is of great significance to find the key genes in the module to study the phenotypic traits controlled by the module [31]. Here, by constructing a gene coexpression network map, key regulatory genes that lead to growth rate differences due to ploidy could be screened out. The size of nodes in the figure represents the connectivity of the node gene, and the larger the node, the higher the connectivity of the gene in the module. The different colors of the nodes represent members of the gene family. Obviously, *PRR5* (pseudo-response regulators 5), *CYP450* (cytochrome P450), and *SPA1* (suppressor of phyA 1) are genes with high connectivity in the turquoise module, and these three genes are related to plant circadian rhythm and may be represent the key genes in this module (Figure 8). It is of great significance to regulate the growth rate differences caused by ploidy differences. Therefore, the hub gene with relatively high connectivity in the coexpression network diagram can be verified by subsequent experiments, which lays a foundation for further analysis and research on the molecular mechanism of slow growth of dwarfed tetraploid.

### 2.8. Circadian Rhythm Is Associated with Ploidy

**Figure 9:**
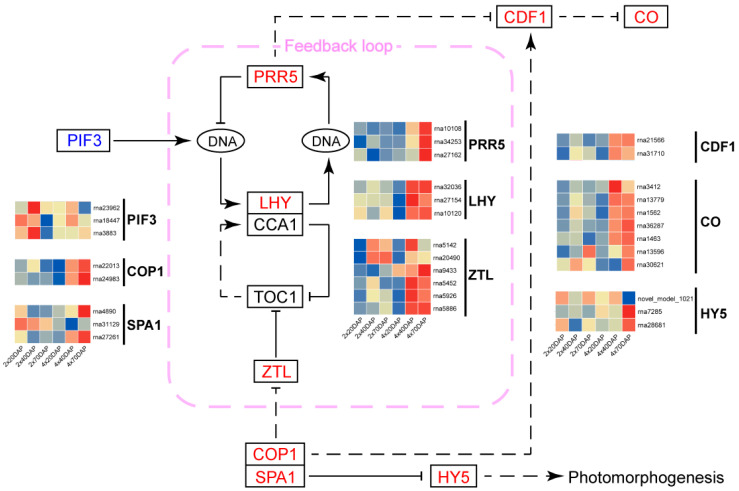
Transcript profiling of genes in the plant circadian pathways. *PIF3*, phytochrome interacting factor 3; *PRR5*, pseudo-response regulators 5; *LHY*, late elongated hypocotyl; *ZTL*, zeitlupe; *COP1*, COP1 E3 ubiquitin ligase; *SPA1*, suppressor of phyA 1; *CDF1*, cycling dof factor 1; *CO*, zinc finger protein constans; *HY5*, elongated hypocotyl 5. The blue gene of the heatmap represents downregulation, while the red gene represents upregulation. Solid lines represent validated regulatory interactions, while dashed lines indicate predicted interactions. An arrow at the end of the line is positively regulated, and a line segment at the end of the line is negatively regulated. The content of the pink dashed box represents the feedback loop.

DEGs of different ploidy in black locust are enriched in the plant circadian rhythm pathways, and the signaling pathways of plant circadian rhythm have been proposed, including the key factors *PIF3*, *PRR5*, *COP1*, and *HY5* [35]. We first identified the genes associated with the plant circadian rhythm pathways in 2× and 4× and found that the differential genes included *PIF3*, *PRR5*, *LHY*, *ZTL*, *COP1*, *SPA1*, *CDF1*, *CO*, and *HY5* (Figure 9). Among them, it is worth noting that *PRR5* and SPA1 were also among the hub genes in the WGCNA analysis (Figure 8). *PIF3* is less expressed in 4× than in 2× and may play a negative regulatory role in circadian rhythms. Except for *PIF3*, most of the remaining genes were upregulated. These results suggest that the differential expression of circadian pathway genes between 2× and 4× may be an important reason for their phenotypic differences.

## 3. Discussion

Chromosome duplication leads to the emergence of diverse phenotypic variations [36]. In this study, the tetraploid black locust ‘D26-5②’ plants and their root length were shorter, and the growth rate was slower than that of diploid black locust ‘D26-5①’, which belonged to the dwarf polyploid type (Figure 2). Dwarfed polyploids, unlike the majority of polyploids, exhibit growth advantages over diploids. However, this situation also occurs in ploidy breeding of other species, such as *populus* and *Citrus wilsonii* (root length of which is shorter than that of diploids), and the growth rate of polyploids of many species is slower than that of diploids [7,37,38]. The study of ploidy variation of this dwarf type will lay a foundation for subsequent understanding of the molecular mechanism of formation and development of dwarf type plants and the cultivation of new varieties of dwarf polyploid, which has a relatively important research value and theoretical significance [39].

The growth rate of plants is regulated by many factors, such as the plants themselves and their environment. Photosynthesis is a process in which plants convert light energy into chemical energy through chlorophyll, which can provide carbon accumulation and energy for plant growth [40]. The reaction rate will significantly affect the growth rate of plants. The time of photosynthesis, photosynthetic efficiency per unit area, and chlorophyll content all affect the photosynthetic efficiency of plants [41]. It has been found that an increase in leaf area after polyploidy indicates an increase in the volume of mesophyll cells and an increase in the content of chloroplasts and chlorophyll in polyploidy, thus increasing the photosynthetic rate [42]. Stomata is an important channel for gas to enter plants. The size and density of stomata per unit area of leaves are closely related to the photosynthetic rate and transpiration of plants. Stomata activity affects plant growth rate by controlling carbon accumulation and water use efficiency [43]. With the decrease in stomatal density and increase in stomatal area, the transpiration rate of plants decreases and the growth rate increases, so the biomass of plants increases [44]. In this study, it was found that the lobular area of the tetraploid ‘D26-5②’ was significantly larger than that of diploid, the lobular thickness was significantly thicker, and the chlorophyll content was also higher than that of the diploid (Figure 3). Stomata density of tetraploid ‘D26-5②’ was significantly lower than that of diploid ‘D26-5①’ black locust (Figure 4). The results showed that the tetraploid ‘D26-5②’ had more advantages in leaf size, chlorophyll content, and stomatal indices, but its growth rate was slower than that of diploid ‘D26-5①’. This phenomenon was found in triploid *papaya* and tetraploid *Ziziphus jujuba* [23,45]. These results indicate that the difference in growth rate may be the main reason for the slow growth of dwarfed tetraploid black locusts.

The results showed that the DEGs could regulate plant growth and development, and change of genetic material led to change of gene expression after chromosome doubling, which may further affect the growth and development of tetraploid ‘D26-5②’ black locusts. Through transcriptome sequencing of diploid ‘D26-5①’ and homologous tetraploid ‘D26-5②’ plants at different developmental stages, DEGs were mainly concentrated in secondary metabolite synthesis, plant hormone signal transduction pathways, and plant circadian rhythm pathways (Figures S4 and S5). Secondary metabolite synthesis are important regulatory pathways of plant growth and development under the condition of homoploidy, but it cannot be explained how they play an important regulatory role leading to slow growth of plants after doubling. Therefore, it is speculated that plant hormone signal transduction and circadian rhythm pathways may be important regulatory pathways leading to relatively slow tetraploid growth. At the same time, weighted coexpression network analysis was used to select the module that was most closely related to ploidy differences, and pathway enrichment was performed on this module’s genes. Most genes were significantly enriched in the plant hormone signal transduction pathway. Moreover, they were significantly enriched in the plant circadian pathway, indicating that the plant hormone signal transduction pathway and the plant circadian pathway may be important factors leading to the observed slow growth of black locust after chromosome doubling (Figure S6). It has also been found and verified that plant endogenous hormones play an important role in plant growth of polyploids in the tetraploid of *Citrus wilsonii* and tetraploid of *populus* [7,38,46].

Plant hormone signal transduction may be an important pathway leading to the slow growth of tetraploid black locust compared to diploid, although this is just a speculation that needs further verification. Therefore, in this study, we sampled diploid ‘D26-5①’ and autotetraploid ‘D26-5②’ black locusts at different developmental stages and determined different endogenous hormone contents. We found different hormone contents in different developmental stages of the two plant types. The results showed that high BR, ETH, SA, ABA, and JA contents in tetraploid ‘D26-5②’ may inhibit the growth of autotetraploid ‘D26-5②’ black locust and make it grow more slowly than the diploid ‘D26-5①’ counterpart (Figure 5). This is in contrast to the results in apple but similar to the findings in *Citrus wilsonii* [7,9]. Although plant endogenous hormones play important roles in the regulation mechanism of slow growth of both tetraploid black locusts and *populus* [47], in tetraploid black locusts, the high content of endogenous hormones that negatively regulate plants leads to dwarfism and slow growth of tetraploid black locusts. The nondetection of significant differences in auxin and CTK contents could possibly be attributed to the fact that these two hormones are not the key factors causing growth disparities in diploid and homologous tetraploid tissue-cultured seedlings within 70 days. Furthermore, the nondetection of gibberellin content in this study could be attributed to the fact that gibberellins may not serve as crucial hormones influencing the development of black locust tissue-cultured seedlings [48]. Polyploid plants grow slowly because of different endogenous hormone contents that promote plant growth [21]. This indicates that the regulation mechanism of endogenous hormones in different plants is also different, and the reasons for this phenomenon still need to be further explored.

Circadian pathways have been shown to correlate with ploidy in mice, but there are fewer relevant studies in plants [49]. In this study, it was found that plant circadian pathway is also a key regulatory pathway of the module closely related to ploidy, and the hub genes in this module—*PRR5*, *CYP450*, and *SPA1*—regulate the plant circadian function (Figure 8). Therefore, it remains to be further explored whether changes in the plant circadian rhythm have an important effect on the growth rate of dwarf tetraploid black locust and how they affect the growth and development of plants. Some studies suggest that the root system of the dwarf black locust is a form of asexual reproduction that enhances its spread in non-native regions, leading to its consideration as an invasive species in some European countries [30]. Future research should focus on the root system architecture of dwarf cultivars, investigating how roots respond to various environmental conditions to form suckers and assessing their potential to invade ecosystems [50]. This line of inquiry is not only crucial for understanding the ecological adaptability of black locust but also has practical implications for developing effective management strategies to control its spread.

## 4. Materials and Methods

### 4.1. Plant Material

The plant material, a diploid black locust ‘D26-5①’ (2×), was sourced from a black locust forest in Miyajiabao, Yanqing District, Beijing, and maintained as aseptic seedlings within our laboratory. The homologous tetraploid black locust ‘D26-5②’ (4×) was obtained by colchicine doubling [9,51]. Plants were kept at 23–25 °C with 16 h light/8 h dark photoperiod, and the shoots of both varieties were subcultured every 40 days in sterile tissue culture flasks containing rooting medium (MS + 0.3 mg·L^−1^ IBA + 30 g·L^−1^ sucrose + 6.5 g·L^−1^ agar) in the base.

### 4.2. Confirmation of Polyploidy

For this, 40-day-old leaves of 2× and 4× tissue culture plantlets were selected, chopped, and fractured with 1.5 mL lysate (9.15 g/L MgCl_2_·6H_2_O, 4.19 g/L 3-(N-morpholino) propansulfonic acid (MOPS), 8.82 g/L sodium citrate, and 0.1% polyethylene glycol p-(1,1,3,3-tetramethylbutyl)-phenyl ether (Triton X-100); pH 7.0) [21]. Nylon cloth was filtered into the sample tube, 10 ug/mL DAPI dye was added into the filtrate for staining, and chromosome ploidy was detected by flow cytometry [52]. Then, shoot tips of plants were cut off between 9:00 and 11:00 in the morning, treated with p-dichlorobenzene for 3 h, dried with filter paper, and fixed in Carnot reagent for 24 h. The fixed shoot tips were washed with distilled water and dissociated with concentrated hydrochloric acid for 15 min. Chromosome number was determined by carbo staining, tablet pressing, and a Cyflow Ploidy Analyzer (Partec, Görlitz, SN, Germany) [53].

### 4.3. Plant Phenotype Determination

The same tissue culture plantlets of 2× and 4× with good growth after 20, 40, and 70 days of subculture were selected. Three groups of biological replicates were performed at each time point, and 15 plants were measured at each biological replicate. ImageJ (National Institute of Mental Health, Bethesda, MD, USA) was used to accurately measure plant height and root length. A functional leaf of each plant was selected from the 15 tissue culture plantlets to be tested in each group. The selected leaves were scanned by a scanner (EPSON ESPERSSION 1680). The photos were used to analyze the leaves’ morphological differences. Paraffin sections were used to observe the leaf thickness [54]. Moreover, after removing the culture medium, plants were weighed with an electronic balance (JJ124BC, Electronic Balance Test Instrument Factory, Changshu, China), and their mass was recorded as fresh weight. After weighing, the plants were placed in tinfoil boxes, then dried at 80 °C to constant weight. Then, they were measured again with electronic balance, and their mass was recorded as dry weight.

### 4.4. Chlorophyll Content Determination

Leaves from 2× and 4× tissue culture plantlets growing for 20, 40, and 70 days after planting (20, 40, and 70 DAP) were weighed and quickly ground with liquid nitrogen. Leaves were placed in a test tube with an appropriate amount of acetone extract (V95% acetone: V5% anhydrous ethanol = 2:1) and placed in 28 °C for 24 h in darkness to fully extract the leaf chlorophyll. Before the determination, the extraction solution was thoroughly mixed. The absorption values of chlorophyll a and chlorophyll b were measured by an ultraviolet spectrophotometer (UV-VIS 1601-PC SHIMADZU, Kioto, Japan) under different wavelength conditions [55]. The maximum absorption peak wavelength was 663 nm for chlorophyll a and 645 nm for chlorophyll b. Three biological replicates per sample were used. The chlorophyll content was calculated as follows:

Chlorophyll a: Chl a = (12.7 × OD663 − 2.69 × OD645) × 0.004 L/0.05 g(1)

Chlorophyll b: Chl b = (22.9 × OD645 − 4.68 × OD663) × 0.004 L/0.05 g(2)

### 4.5. Histological Observations

Three functional leaves with stable growth were randomly selected from the 2× and 4× tissue culture plantlets at 20, 40, and 70 DAP. Transparent nail polish was applied to the back of the leaves, and after a moment, the nail polish film was torn off and spread on a slide. Then, the stomata morphology was observed with confocal microscopy (model LSM 710; Carl Zeiss, https://www.zeiss.com (accessed on 19 January 2024) [56]. Photographs were then taken for the upper, lower, left, right, and middle regions of each visual field, and ImageJ was then used to measure stomatal length, width, and density.

### 4.6. Plant Endogenous Hormone Content Determination

The endogenous coenzymes, gibberellins (GA), cytokinins (CTK), abscisic acid (ABA), ethylene (ETH), brassinosteroids (BR), jasmonic acid (JA), and salicylic acid (SA) were detected using LC–MS analysis on the AB Sciex QTRAP6500 LC–MS/MS platform by Metware Metabolomics (http://www.metware.cn/ (accessed on 19 January 2024) [57,58]. The fresh 0.5 g samples of 2× and 4× whole tissue culture plantlets, which were cultured in root medium for 20, 40, and 70 days, were rapidly frozen in liquid nitrogen and subsequently ground into powder using a mortar for subsequent determination of the aforementioned endogenous hormone contents.

### 4.7. Transcriptome Sequencing, Function Annotation, and Differentially Expressed Genes

RNA samples of 2× and 4× tissue culture plantlets were extracted at 20, 40, and 70 DAP using a TransZol Up Plus RNA Kit (Tiangen, Shanghai, China). Intense RNA bands were shown through agarose gel electrophoresis, and a NanoDrop 2000 spectrophotometer (Thermo Scientific, MA, USA) detected ratios of ≥2 and 1.8 for A260/A230 and A260/A280, respectively [59]. Sequencing using the Illumina HiSeq X Ten platform (Illumina, San Diego, CA, USA) was performed at IGENECODE, Beijing, China. The sequencing of libraries from each sample was carried out with three biological replicates. After filtering out adapter and low-quality reads from the raw data, the clean reads were aligned to the genome using the HISAT v 2.04 software with default parameters [60]. Q20 and Q30 are quality scores that measure the percentage of bases with sequencing error rates of less than 1% and 0.1%, respectively. Higher Q20 and Q30 scores indicate better sequencing quality and accuracy [61]. The transcript expression levels were normalized based on fragments per kilobase of transcript sequence per million base pairs sequenced (FPKM). Differentially expressed genes (DEGs) between 2× and 4× tissue culture plantlets at different developmental stages were identified using the DESeq2 R package (1.10.1) [62]. The thresholds for DEGs were set to an adjusted padj < 0.05 and |log_2_(fold change)| ≥ 1. The enrichment analysis methods of GO (Gene Ontology) and KEGG (Kyoto Encyclopedia of Genes and Genomes) were used, and the enrichment GO entries and the biological pathways involved were learned jointly [63].

### 4.8. Identification of Differentially Expressed Genes by qRT-PCR

Transcriptions of RNA extracted from 2× and 4× tissue culture plantlets at different developmental stages were performed by the Fast Quant Reverse Transcription System (Tiangen, Shanghai, China). Then, the SuperReal PreMix Plus Kit (SYBR Green) was used for real-time fluorescence quantitative PCR detection after prime 5 website was used to design primers for real-time quantitative analysis of 14 DEGs (Table S2). These 14 DEGs related to growth and development in 2× and 4× black locusts were randomly selected from the bHLH, AP2-ERF, and GRAS families [64].

### 4.9. Weighted Gene Coexpression Network Analysis (WGCNA)

The differentially expressed genes of 2× and 4× were analyzed by WGCNA [65]. Samples and genes that do not conform to the quality were eliminated by the algorithm. After filtering, correlation between genes was calculated, the similarity between genes was determined, and the *β* value was determined. Then, the expression relationship between genes was calculated, and the topological matrix was established according to the TOM value. Sample clustering was conducted to cluster the samples into different groups. After clustering, the R language algorithm was used for hierarchical clustering. The hierarchical clustering tree was constructed according to the gene expression, minimum number of genes in the module, and minimum distance of the merging modules. The genes were divided into different modules. The same module had high gene correlation and similar biological functions. Principal component analysis was performed on all genes in the module, and the first principal component value represented the overall gene expression level of the module, which was called module eigengene or module eigengene (ME).

The approach adopted in this study was to screen key modules by phenotypic association. The correlation coefficient between the gene expression level and the dependent variable level was calculated as gene significance (GS) and module significance (MS) so as to determine the degree of association between the module and the phenotype. Then, GO and KEGG enrichment analyses were carried out to determine the main biological process of gene enrichment in the selected module. Key module genes were selected to construct the gene coexpression network map, and key regulatory genes in the module were screened. The output notes and edge files of WGCNA were imported, and a control network diagram was constructed according to the weight value (TOM value). The hub gene with high connectivity in this module was screened, which was generally located in the upstream of the regulatory network and was the key gene [66].

## 5. Conclusions

Compared to diploids, we found that the growth rate of tetraploid black locust was significantly slower, both in terms of plant height and root length. Additionally, the chlorophyll content of the tetraploids was greater than that of the diploids throughout the growth cycle, indicating their high photosynthetic capacity. Our data further showed that the BR, ETH, SA, ABA, and JA contents in the tetraploids were greater than those in the diploids. In particular, BR contents in the tetraploids were highly significant at 20, 40 and 70 days of development. A module closely related to black locust ploidy was identified by transcriptome sequencing and WGCNA analysis, in which *PRR5*, *CYP450*, and *SPA1* were identified as the hub genes in the network. Through transcriptome analysis, we also identified the plant circadian pathway as an important pathway, and the majority of genes in this pathway were expressed at a higher rate in tetraploids than in diploids. This is the first time that metabolite analysis and comparative transcriptomics were carried out in 2× and 4× black locusts, and the network to ploidy was preliminarily explored. We have provided guidance for further studies on the role of hub genes in response to polyploid breeding of black locust and even woody plants.

## Supplementary Materials

The following supporting information can be downloaded at: https://www.mdpi.com/article/10.3390/ijms25021312/s1.

## Abbreviations

2×, diploid; 4×, homologous tetraploid; BR, brassinosteroid; DEGs, differentially expressed genes; WGCNA, weighted gene coexpression network analysis; *PRR5*, pseudo-response regulators 5; *CYP450*, cytochrome P450; *SPA1*, suppressor of phyA 1.
