# Supplementary material for: Transcriptomic and Phenotypic Analyses Reveal the Molecular Mechanism of Dwarfing in Tetraploid Robinia pseudoacacia L
Source: Int J Mol Sci. 2024 Jan 21;25(2):1312. doi: 10.3390/ijms25021312 (PMC10816058; doi:10.3390/ijms25021312)
Supplement: Supplementary file 1 [file ijms-25-01312-s001.zip › ijms-2800731-supplementary/Supplementary Figure-legend .pdf]

Supplementary Figure S1. Comparison of phenotypic indexes for 2x and 4x black locust tissue culture plantlet 6 months of transplantation. Scale: 10cm.

Supplementary Figure S2. Comparison of endogenous IAA and TZ content between 2x and 4x black locust at different developmental stages. * represents significant difference (* $P<0.05$); ** represents highly significant difference (** $P<0.01$).

Supplementary Figure S3. qRT-PCR validation of differential genes in 2x and 4x at different developmental stages.

Supplementary Figure S4. GO enrichment of differentially expressed genes in 2x and 4x black locust at different developmental stages. **(A)** 20DAP. **(B)** 40DAP. **(C)** 70DAP.

Supplementary Figure S5. KEGG enrichment of differentially expressed genes in 2x and 4x black locust at different developmental stages. **(A)** 20DAP. **(B)** 40DAP. **(C)** 70DAP.

Supplementary Figure S6. Turquoise module gene GO and KEGG enrichment analysis. **(A)** GO enrichment. **(B)** KEGG enrichment.

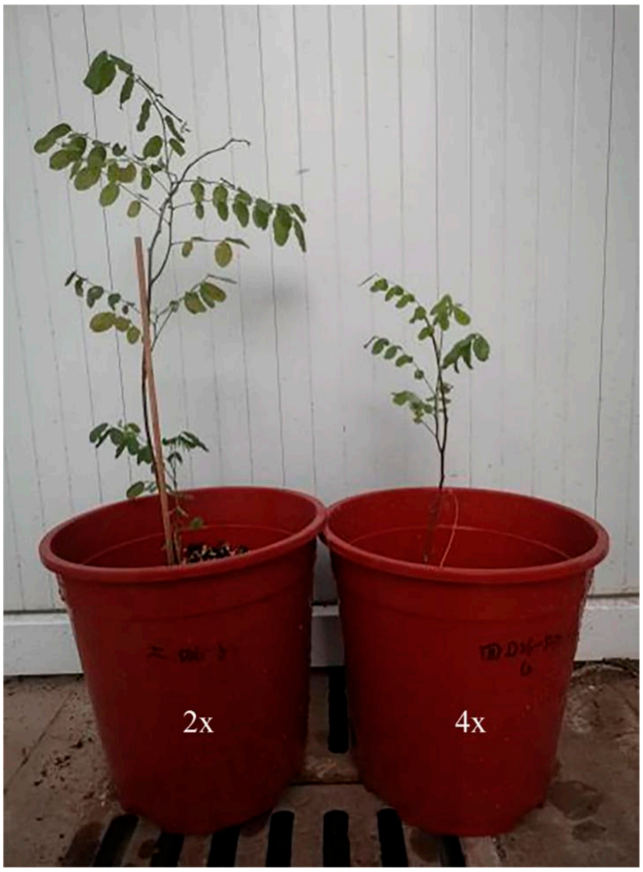

Supplementary Figure S1. Comparison of phenotypic indexes for 2x and 4x black locust tissue culture plantlet 6 months of transplantation. Scale: 10cm.

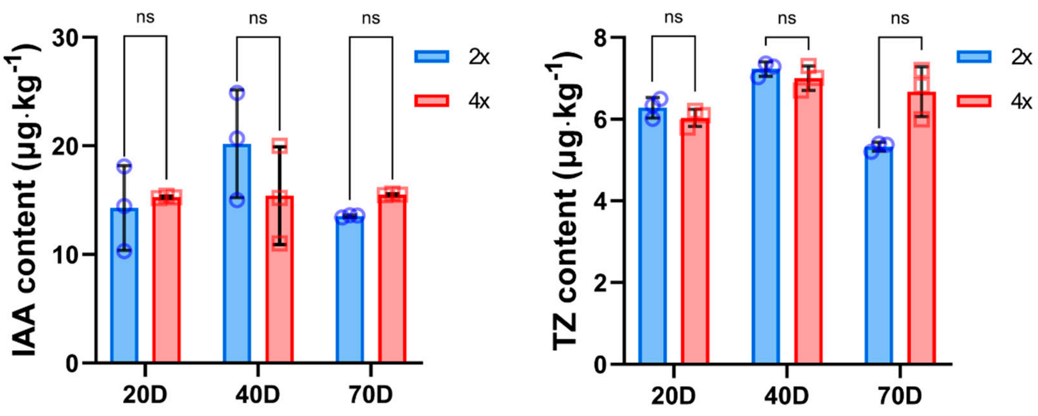

Supplementary Figure S2. Comparison of endogenous IAA and TZ content between 2x and 4x black locust at different developmental stages. * represents significant difference (* P<0.05); ** represents highly significant difference (** P<0.01).

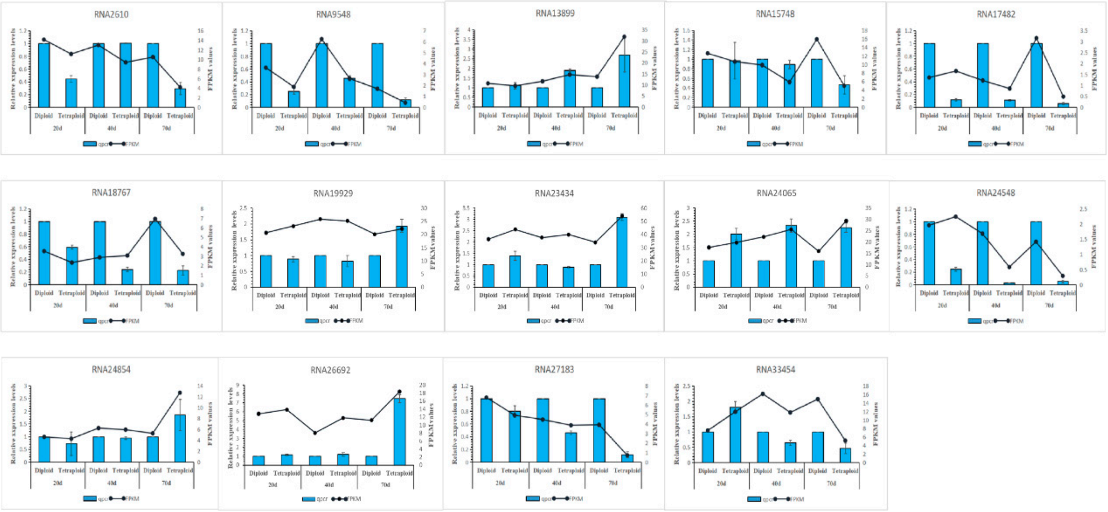

Supplementary Figure S3. qRT-PCR validation of differential genes in 2x and 4x at different developmental stages.

A

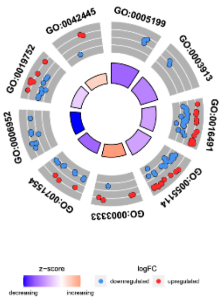

ID	Description
GO:0005199	structural constituent of cell wall
GO:0003913	DNA photolyase activity
GO:0016491	oxidoreductase activity
GO:0055114	oxidation−reduction process
GO:0003333	amino acid transmembrane transport
GO:0071554	cell wall organization or biogenesis
GO:0006952	defense response
GO:0019752	carboxylic acid metabolic process
GO:0042445	hormone metabolic process

C

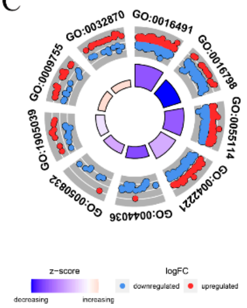

ID	Description
GO:0016491	oxidoreductase activity
GO:0016798	hydrolase activity, acting on glycosyl bonds
GO:0055114	oxidation−reduction process
GO:0042221	response to chemical
GO:0044036	cell wall macromolecule metabolic process
GO:0050832	defense response to fungus
GO:1905039	carboxylic acid transmembrane transport
GO:0009755	hormone−mediated signaling pathway
GO:0032870	cellular response to hormone stimulus

B

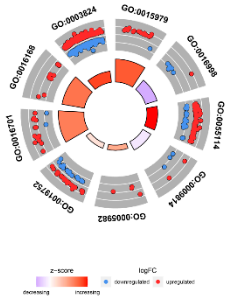

ID	Description
GO:0015979	photosynthesis
GO:0016998	cell wall macromolecule catabolic process
GO:0055114	oxidation−reduction process
GO:0009814	defense response, incompatible interaction
GO:0005982	starch metabolic process
GO:0019752	carboxylic acid metabolic process
GO:0016701	oxidoreductase activity, acting on single donors with incorporation of molecular oxygen
GO:0016168	chlorophyll binding
GO:0003824	catalytic activity

Supplementary Figure S4. GO enrichment of differentially expressed genes in 2x and 4x black locust at different developmental stages. **(A)** 20DAP. **(B)** 40DAP. **(C)** 70DAP.

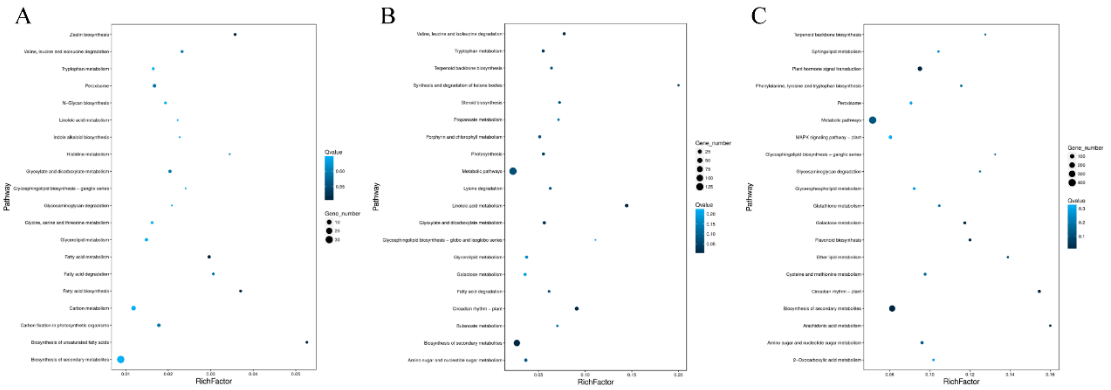

Supplementary Figure S5. KEGG enrichment of differentially expressed genes in 2x and 4x black locust at different developmental stages. **(A)** 20DAP. **(B)** 40DAP. **(C)** 70DAP.

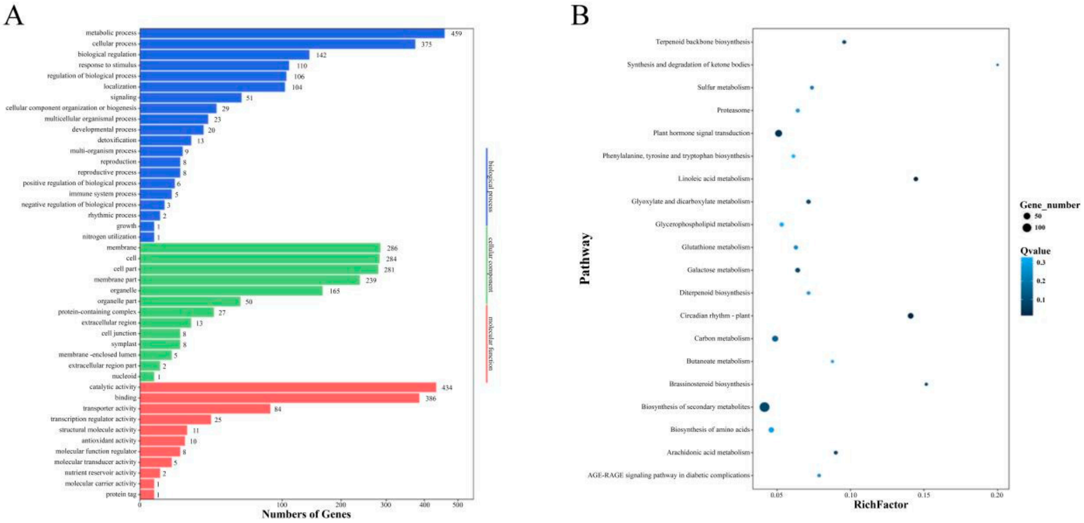

Supplementary Figure S6. Turquoise module gene GO and KEGG enrichment analysis. **(A)** GO enrichment. **(B)** KEGG enrichment.
