# Supplementary figures and images for: Transcriptomic and Phenotypic Analyses Reveal the Molecular Mechanism of Dwarfing in Tetraploid Robinia pseudoacacia L
Source: Int J Mol Sci. 2024 Jan 21;25(2):1312. doi: 10.3390/ijms25021312 (PMC10816058; doi:10.3390/ijms25021312)

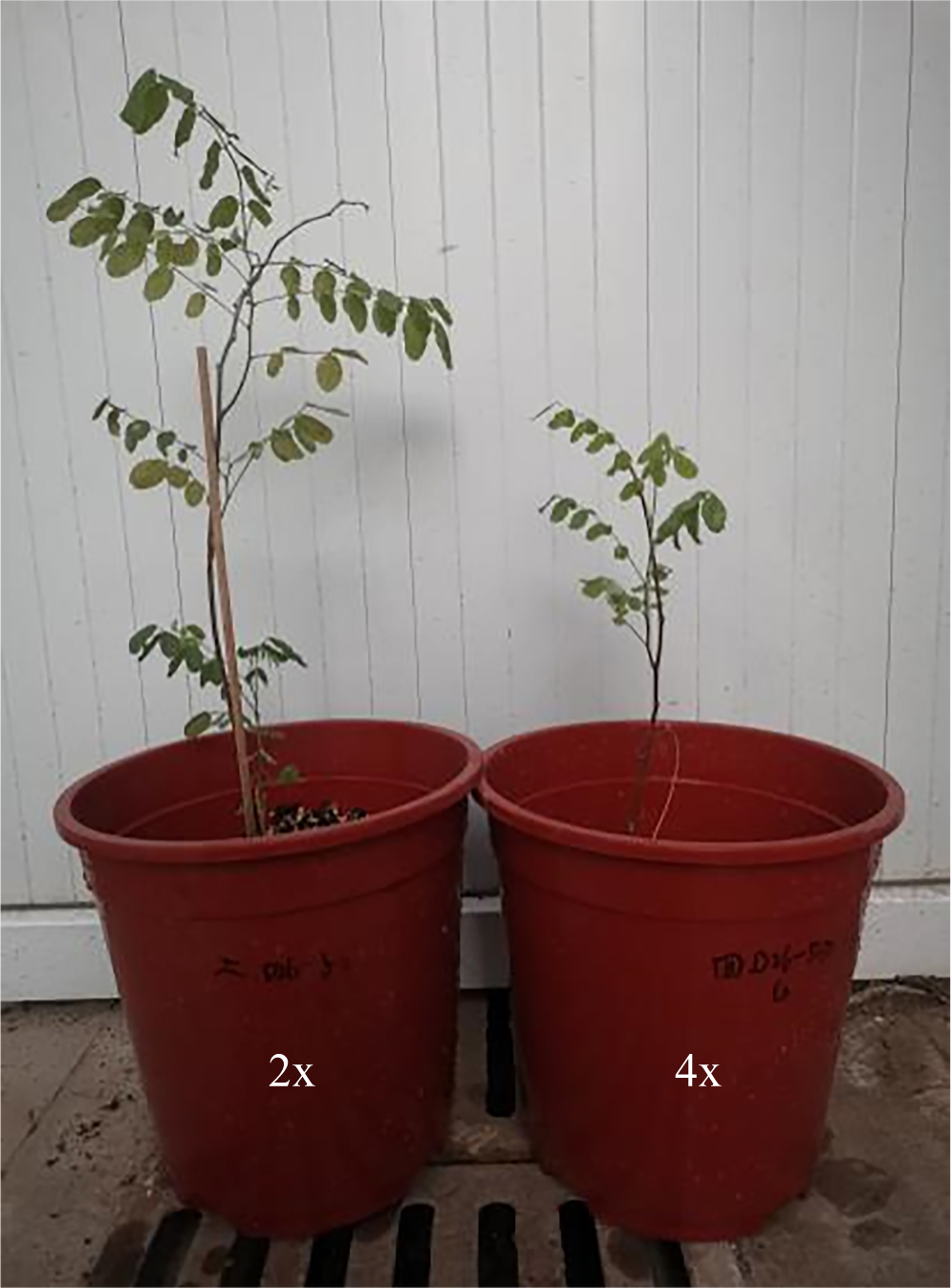

Supplement: Supplementary file 1 [file ijms-25-01312-s001.zip › ijms-2800731-supplementary/Supplementary Figure 1.tif]

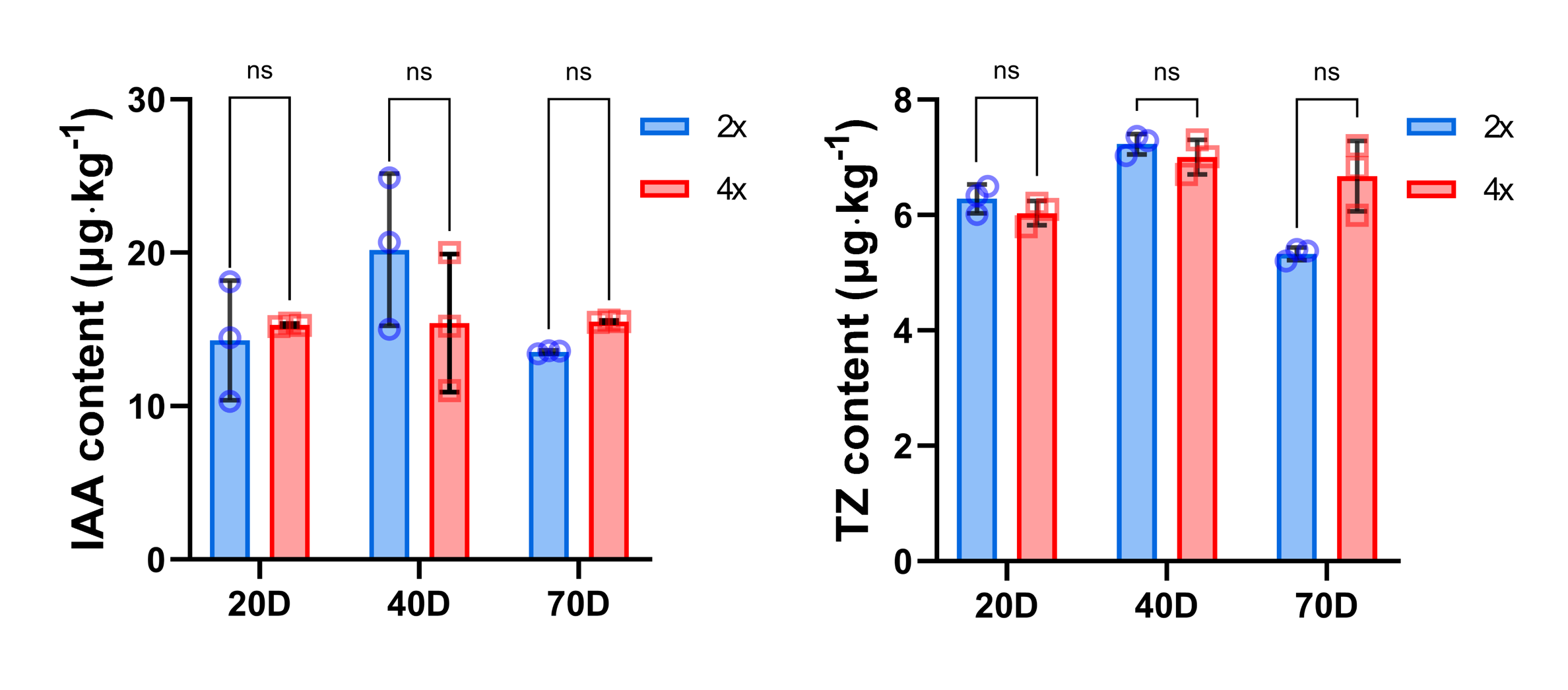

Supplement: Supplementary file 1 [file ijms-25-01312-s001.zip › ijms-2800731-supplementary/Supplementary Figure 2.tif]

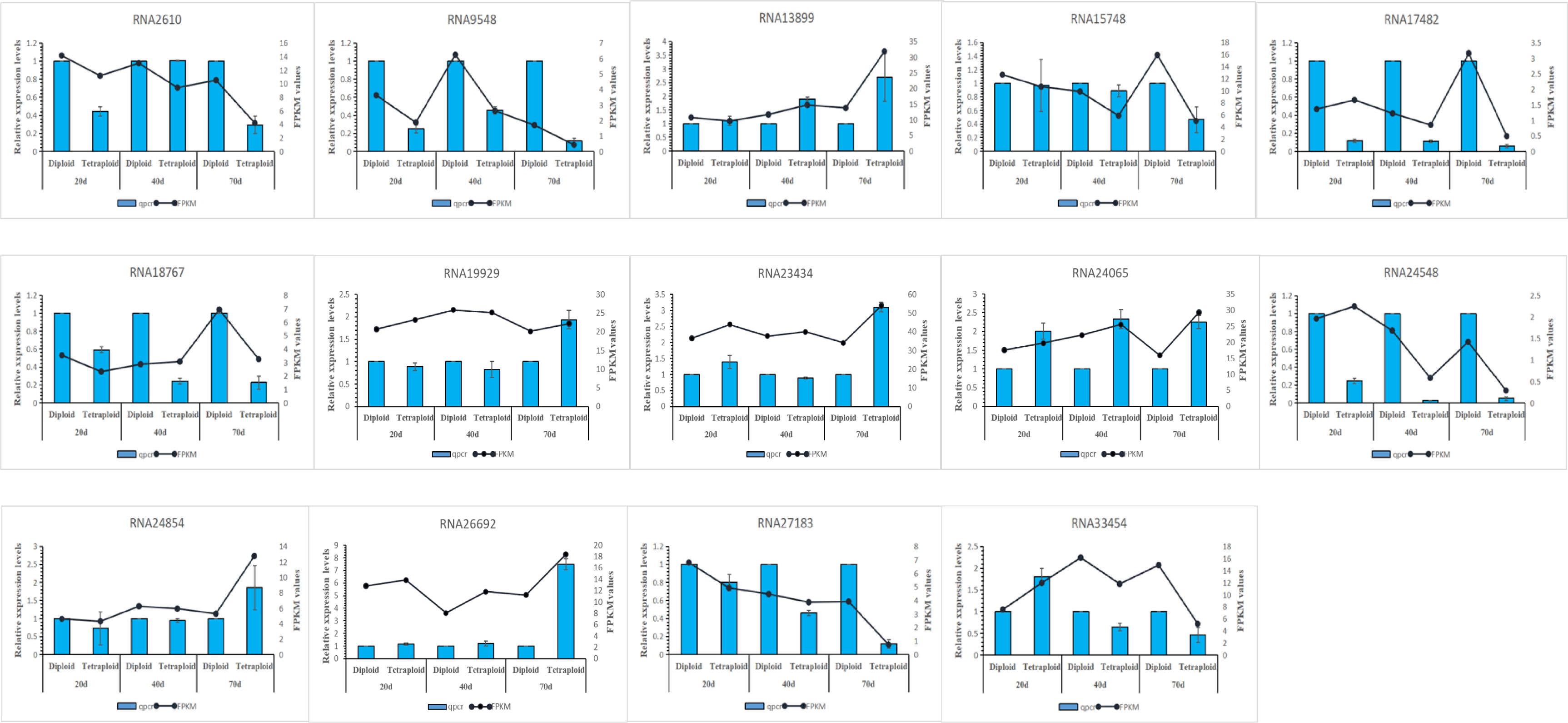

Supplement: Supplementary file 1 [file ijms-25-01312-s001.zip › ijms-2800731-supplementary/Supplementary Figure 3.tif]

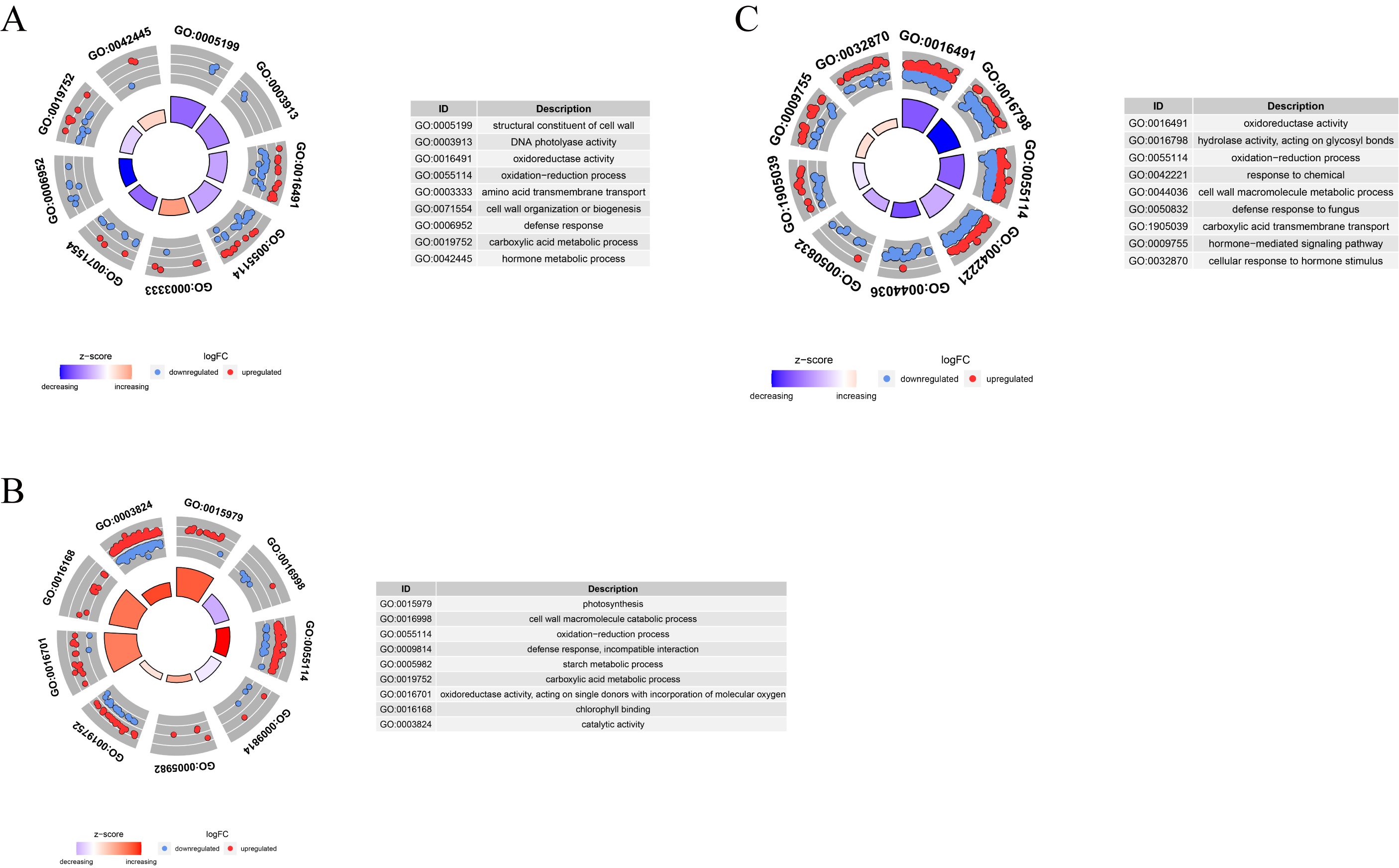

Supplement: Supplementary file 1 [file ijms-25-01312-s001.zip › ijms-2800731-supplementary/Supplementary Figure 4.tif]

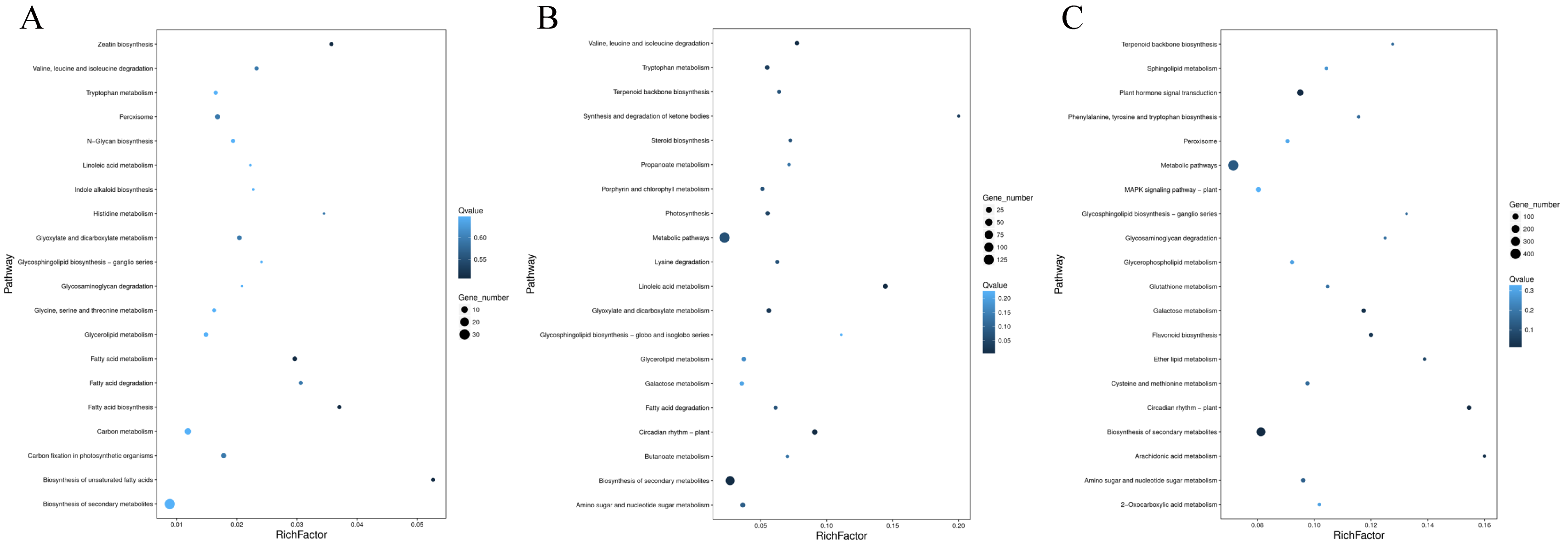

Supplement: Supplementary file 1 [file ijms-25-01312-s001.zip › ijms-2800731-supplementary/Supplementary Figure 5.tif]
